# A Review on Microfluidic Paper-Based Analytical Devices for Glucose Detection

**DOI:** 10.3390/s16122086

**Published:** 2016-12-08

**Authors:** Shuopeng Liu, Wenqiong Su, Xianting Ding

**Affiliations:** Institute for Personalized Medicine, School of Biomedical Engineering, Shanghai Jiao Tong University, Shanghai 200030, China; spliu613@sjtu.edu.cn (S.L.); suwenqiong@sjtu.edu.cn (W.S.)

**Keywords:** microfluidic paper-based analytical devices (μPAD), glucose, colorimetric detection, electrochemical detection

## Abstract

Glucose, as an essential substance directly involved in metabolic processes, is closely related to the occurrence of various diseases such as glucose metabolism disorders and islet cell carcinoma. Therefore, it is crucial to develop sensitive, accurate, rapid, and cost effective methods for frequent and convenient detections of glucose. Microfluidic Paper-based Analytical Devices (μPADs) not only satisfying the above requirements but also occupying the advantages of portability and minimal sample consumption, have exhibited great potential in the field of glucose detection. This article reviews and summarizes the most recent improvements in glucose detection in two aspects of colorimetric and electrochemical μPADs. The progressive techniques for fabricating channels on μPADs are also emphasized in this article. With the growth of diabetes and other glucose indication diseases in the underdeveloped and developing countries, low-cost and reliably commercial μPADs for glucose detection will be in unprecedentedly demand.

## 1. Introduction

Glucose, one of the essential metabolic intermediates, is an important medical analyte which is the indicator of various diseases, such as glucose metabolism disorders and islet cell carcinoma [1,2,3,4]. Normally, the concentration of glucose in human blood stream is in the range of 3.8–6.9 mM. A level below 2.8 mM after no-eating or following exercise is considered to be hypoglycemia [5]. For diabetics, the blood glucose concentration should be strictly controlled below 10 mM according to the American Diabetes Association [6]. Frequent and convenient monitor of the blood glucose concentration is a key endeavor for medical diagnosis [7,8] and of critical importance to the diabetics for the hyperglycemia complications prevention [9,10,11,12].

A terminology “ASSURED” representing the words “affordable, sensitive, specific, user-friendly, rapid and robust, equipment-free and delivered to those in need”, is summarized by the World Health Organization (WHO) as the guidelines for the diagnostic point-of-care tests (POCTs) [13]. These diagnostic tests are emerging for applications in the underdeveloped and developing world, where cost-effect and simplicity are of major concerns [14,15,16,17]. As the most abundant biopolymer on the Earth, cellulose is mostly used to produce paper for industrial use. Being composed of a network of hydrophilic cellulose fibers [18], paper has a natural porous microstructure, which is amenable to lateral flow via capillary action, realizing on-site analysis without the requirement for external forces such as pumps [14,19].

Microfluidic paper-based analytical devices (μPADs) as a promising and powerful platform have shown great potential in the development of POCTs [20,21,22,23,24]. This concept was first proposed by the Whitesides group in 2007 [14] and the photoresist-patterned paper was used to fabricate the microfluidic devices that the liquid could transport through capillary force in the lack of external equipment. Since then, μPADs have been popular in a variety of applications, such as clinical diagnostics [13,14,25,26,27,28,29,30], food safety [31,32], environmental monitoring [33,34,35] and bioterrorism [36,37,38,39,40] due to the advantages of portability, simplicity, economic affordability and minimal sample consumption. 

Paper substrate is hydrophilic by nature. Therefore, to fabricate the μPADs, hydrophobic barriers are usually created to confine the fluid flow within a desired location or direct the fluidics follow desired trails. A number of techniques, including photolithography [14,41,42,43,44,45,46], wax printing [47,48,49,50], screen-printing [51,52], plasma treating [53,54], flexography [55,56,57] and laser treating [58] have been developed for the manufacture of hydrophobic barriers. In the photolithography process, photoresists, e.g., octadecyltrichlorosilane (OTS), poly(*o*-nitrobenzylmethacrylate) (PoNBMA) and SU-8 used to fabricate μPADs are costly and the expensive photolithography equipment is also required. Patterning paper with wax printing technology could offer relative high speed, facile process and high resolution for fabricating μPADs, while the commercial wax printers of high running costs and the wax of low melting point restrict the use in batch production. Screen-printing method exhibits slightly higher resolutions than wax printing, but it is limited by the requirements of accordingly various printing screens when patterns are changed. Although plasma treating produces patterns without affecting their flexibility or surface topography, this method suffers from the limitation of mass production. Flexographic printing is considered as a proper technique for mass production. However, its requirements locate at the two prints of polystyrene and different printing plates. High resolution could be achieved when fabricating μPADs using laser treating method, but it is of difficulties to fold or store the laser-treated devices [59,60]. Though each fabrication method has its own advantages and limits, the economic benefit of μPAD mass production is the principal issue in concerned, especially for the widespread utilization in glucose detection. Balancing the interests between cost and performance may rely on the development of unique process technology and new materials.

With the development of μPADs, multiple conventional detection techniques, such as colorimetric detection [59,61,62,63,64], electrochemical detection [65,66,67,68], chemiluminescence (CL) [69,70,71,72,73], fluorescence [74,75,76,77], mass spectrum (MS) [78,79] and surface-enhanced Raman spectroscopy (SERS) [80,81] have been applied to paper-based devices for rapid diagnostics.

In this article, colorimetric and electrochemical μPADs for glucose detection in the past five years are summarized and reviewed. With the development of microfabrication and nanomaterial, glucose detection μPADs with high sensitivity and stability will be commercially accessible in the near future.

## 2. Colorimetric Detection of Glucose

### 2.1. Fabrication Process of Colorimetric Glucose μPADs

**Figure 1:**
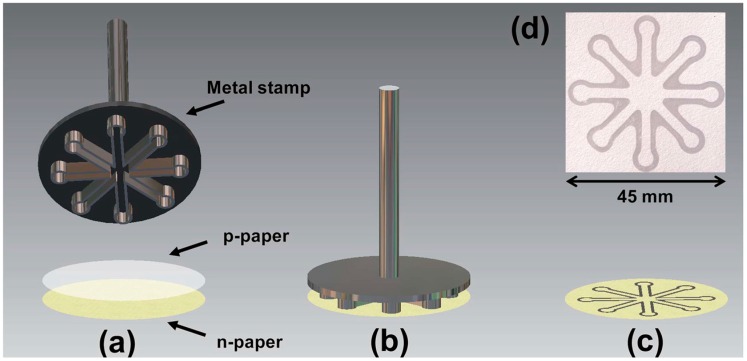
Scheme of the typical stamping process for microfluidic paper-based analytical devices (μPADs) fabrication: (**a**) a native paper (n-paper) is covered by a paraffinized paper (p-paper); (**b**) after heated at 150 °C, the metal stamp is pressed against the layered paper pieces; (**c**) a typical μPAD fabricated by the stamping process and its optical micrograph (**d**). With the permission from [62]; Copyright 2014, The Royal Society of Chemistry.

**Figure 2:**
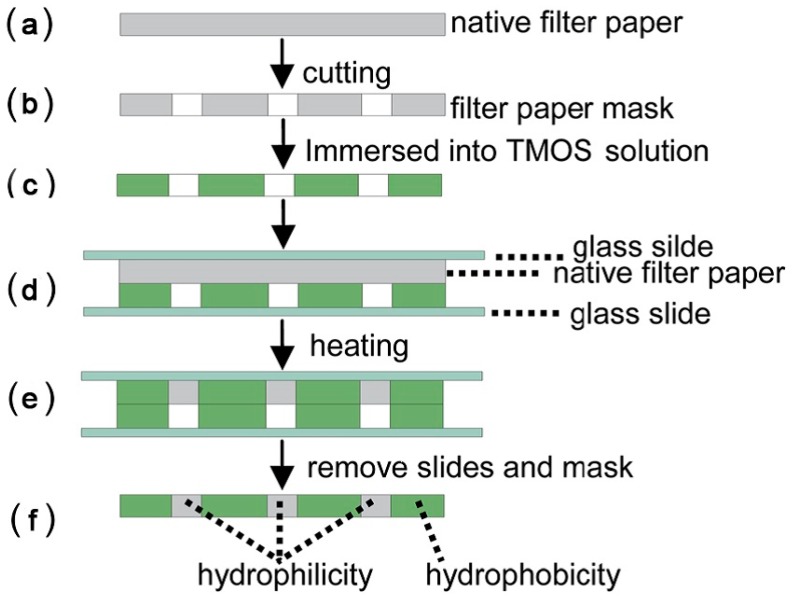

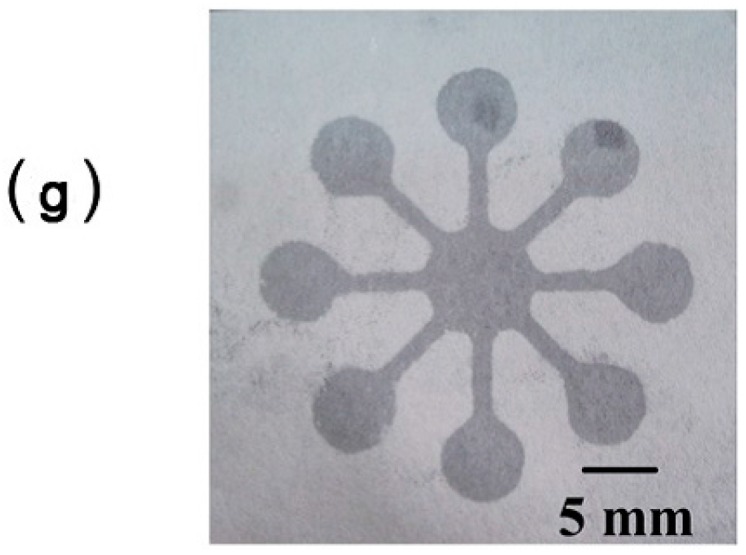
Scheme of the μPAD fabrication in [59]: A filter paper mask (**b**) was obtained by cutting on a native filter paper (**a**), and was immersed in TMOS solution (**c**); The TMOS-adsorbed mask and a native filter paper were packed between two glass slides (**d**); TMOS molecules were assembled on the native filter paper by heating (**e**); and the fabricated μPAD with hydrophilic-hydrophobic contrast (**f**) and its photograph (**g**) obtained by spraying water on it. With the permission from [59]; Copyright 2014, The Royal Society of Chemistry.

**Figure 3:**
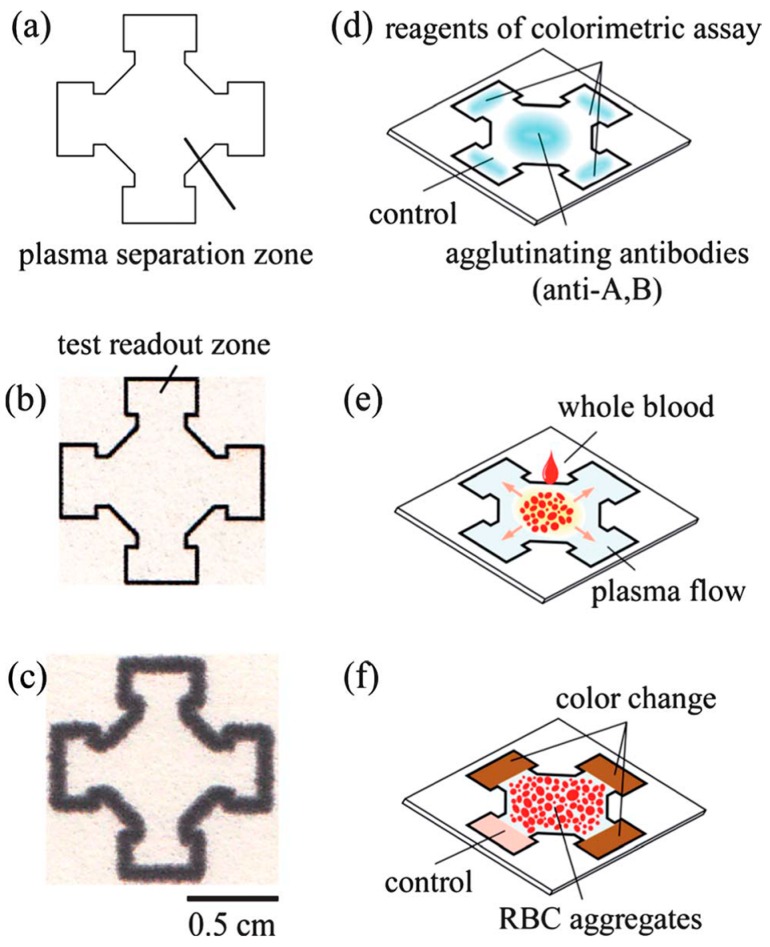
Fabrication scheme of the μPAD designed in [91]. The central plasma separation zone (**a**) and the four test readout zones (**b**) were patterned on chromatography paper by a wax printer (**c**); (**d**) Agglutinating antibodies were immobilized at the central part while the reagents for the colorimetric assay at the periphery zones; (**e**) To perform a diagnostic test with the developed μPAD, the whole blood sample was dropped onto the plasma separation zone; (**f**) The red blood cells were agglutinated in the central zone, while the separated plasma wicked into the test readout zones and reacted with the reagents of the colorimetric assay. With the permission from [91]; Copyright 2012, The Royal Society of Chemistry.

Colorimetric detection has been the most widely employed technique for paper-based analytical devices due to the advantages of visual readout, straightforward operation and superior stability [82,83,84,85]. Glucose oxidase (GOx) and horseradish peroxidase (HRP) are the commonly used bienzyme system to catalyze the reaction between glucose and the color indicator in μPADs. The catalytic reaction of glucose by glucose oxidase results in hydrogen peroxide (H_2_O_2_) and gluconic acid. Peroxidase then catalyzes the reaction of H_2_O_2_ with color indicator and generates a visual color change. Identifying an appropriate color indicator is one of the crucial steps in the advancement of μPADs for the glucose concentrations determination. Potassium iodide (i.e., KI) was one of the commonly used color indicators. HRP catalyzes the oxidation of iodide to iodine by hydrogen peroxide, leading to a change from colorless to a visual brown color [59,60,62,86,87,88,89,90,91]. Garcia et al. [62] proposed a production method of μPAD using a handheld metal stamp (Figure 1). The channel and barrier widths of the fabricated μPAD were 2.6 ± 0.1 and 1.4 ± 0.1 mm, respectively. The improvement in the color uniformity was created by the covalent coupling of enzymes on the surface of paper. The linear response was in the range from 0 to 12 mM. Cai et al. [59] developed a μPAD fabricated free of metal masks or expensive equipment. A mask immobilized with trimethoxyoctadecylsilane (TMOS) was used to silanize the cellulose paper substrate by heating the paper, which was located between the mask and glass slides. TMOS adsorbed on the mask would evaporate and penetrate into the cellulose paper aligning onto the mask, while other parts remained hydrophilic due to the lack of reaction between cellulose OH groups and TMOS (Figure 2). Li et al. [60,86] developed a piezoelectric ceramic transducer (PZT) drop-on-demand wax droplet generating system for μPADs. Wax was jetted as droplet and shaped to form the hydrophobic fluid pattern on a piece of filter paper with a PZT actuator. Mohammadi et al. [88] proposed a screen-printing method to fabricate μPAD through patterning polydimethylsiloxane (PDMS) instead of wax onto paper to construct hydrophilic channels. The glucose diagnostic device could be developed by drawing with a silane/hexane ink without further requirement of complex equipment. Oyola-Reynoso et al. [92] used a ball-point pen in the fullness of a solution of trichloro perfluoroalkyl silane in hexanes to draw hydrophobic regions of paper. To investigate the glucose concentration in blood plasma, Yang et al. [91] developed a μPAD with agglutinating antibodies immobilized for separating blood plasma from red blood cells in whole blood (Figure 3). Furthermore, laser-induced photo-polymerisation [93] and blade coating [64] were also used for creation of μPADs depending on GOx/HRP bienzyme reaction.

### 2.2. Alternative Color Indicators for Glucose μPADs

**Figure 4:**
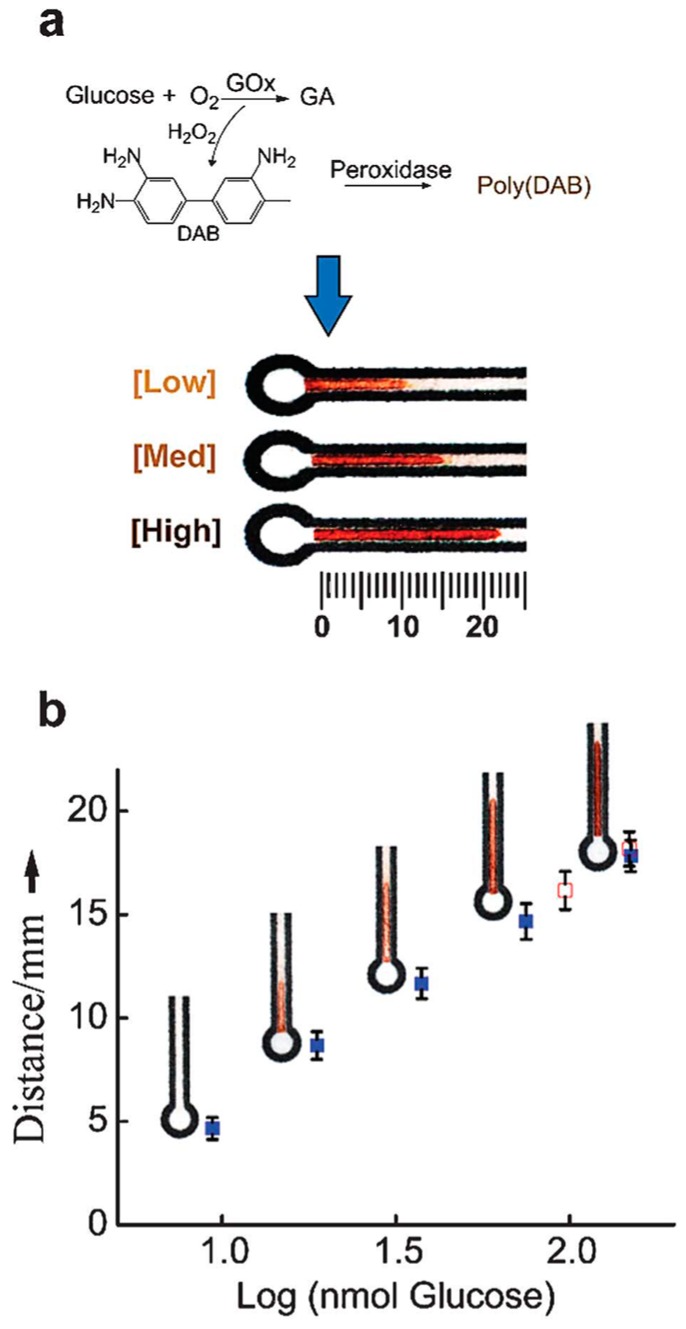
Scheme of the μPAD fabrication in [35] (**a**) Poly(DAB) as a brown precipitate was generated in the present of peroxidase when DAB reacted with H_2_O_2_ which came from the oxidation of glucose under the existing of glucose oxidase. By the capillary effect, the brown distance along the channels was related to the concentration of glucose involved in the reactions; (**b**) The standard calibration curves (closed blue squares) of the color development distance with the standard glucose solutions. The real (complex) serum samples (opened squares) contained 100 nM glucose according to the color development distance. With the permission from [35]; Copyright 2013, The Royal Society of Chemistry.

Due to the weaker color signal produced by potassium iodide, some organics and nanoparticles were used as color indicators in glucose μPADs. 2,4,6-tribromo-3-hydroxy benzoic acid (TBHBA) and 4-aminoantipyrine (4-APP) were used as substrates catalyzed by HRP to generate color signal for glucose detection due to superior water solubility of TBHBA and positive charges of TBHBA/4-APP which can be attached firmly onto paper substrate with negative charges [94,95]. Chen et al. [4] replaced TBHBA with *N*-ethyl-*N* (3-sulfopropyl)-3-methyl-aniline sodium salt (TOPS) and used TOPS/4-APP in μPAD for glucose detection, which showed a limit of detection (LOD) of 38.1 μM. Gabriel et al. [96] used 4-AAP and sodium 3,5-dichloro-2-hydroxy-benzenesulfonate (DHBS) as the chromogenic solution. Chitosan was involved to improve the sensing performance of glucose in tear samples and the detection limit was 0.023 mM. Zhou et al. [61] used cross-linked siloxane 3-aminopropyltriethoxysilane (APTMS) as probe for colorimetric μPAD. Only glucose oxidase needs to be immobilized on the μPAD due to a visual color change when APTMS/glutaraldehyde (GA) complex reacted with H_2_O_2_. The μPAD exhibited good linearity for the concentration in the range from 0.5 to 30 mM, covering the clinical range for normal blood glucose level [6]. Similarly, Soni et al. [97] used co-immobilized color pH indicator for direct determination of salivary glucose with no need for peroxidase. While most conventional intensity-based colorimetric μPAD were still constrained to the requirement of camera for quantitative detection, Cate et al. [35] and Wei et al. [63] utilized visual distance-based methods for μPADs through the distance of color development as a detection value. GOx and colorless 3,3′-diaminobenzidine (DAB) were immobilized in a hydrophilic channel as the substrate on the μPADs. H_2_O_2_ were generated by GOx when sample solution travelled along the channel by capillary action, and then further reacted with DAB to form a visible brown, insoluble product (poly(DAB)) in the presence of peroxidase (Figure 4). The length of the brown precipitate was positively correlated to the concentration of glucoses.

**Figure 5:**
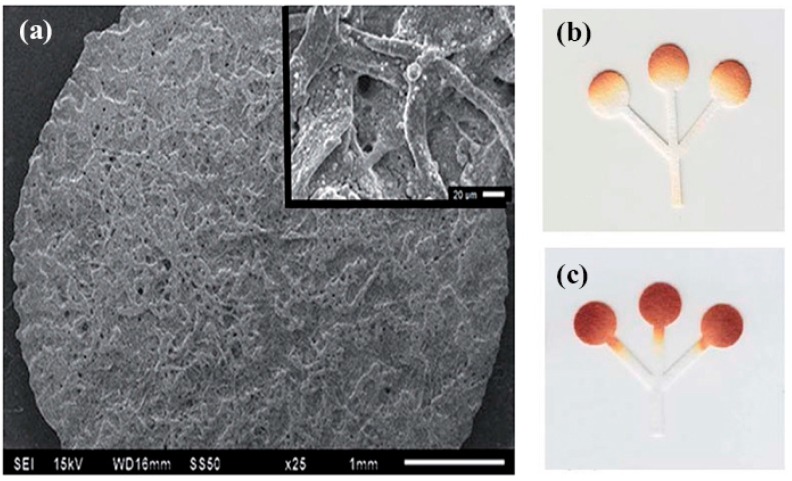
(**a**) Scanning electron microscope (SEM) images of the μPAD in [100] after the SiO_2_ nanoparticles deposition. Optical images of the μPADs with (**c**) and without (**b**) SiO_2_ nanoparticles modification applied to the colorimetric assay for glucose. Adapted with the permission from [100]; Copyright 2014, The Royal Society of Chemistry.

Nanoparticles have been used in lateral flow assays associated with colorimetric detection to improve the analytical performance and minimize washing effects [98,99,100]. Figueredo et al. [99] applied three different types of nanomaterials, namely Fe_3_O_4_ nanoparticles (MNPs), multiwalled carbon nanotubes (MWCNT), and graphene oxide (GO) in paper-based analytical devices to improve the homogeneity on color measurements. Instead of constructing hydrophobic barriers on paper surface as described above, a layer of hydrophilic paper channels was directly built up on the surface of a hydrophobic substrate. With the assistance of glucose oxidase and HRP, the LOD of the μPADs treated with MNPs, MWCNT and GO were 43, 62, and 18 μM, respectively. Evans et al. [100] also aimed at improving color intensity and uniformity by using silica nanoparticles (Figure 5). The PAD added with silica nanoparticles can prevent the color gradients in the colorimetric detection caused by the washing away effect and the LOD was 0.5 mM. According to the ability of glucose oxidase to reduce Au^3+^ ions to Au^0^ in the presence of glucose [101,102], Palazzo et al. [98] used gold nanoparticles (AuNPs) as colorimetric reporters to detect glucose. This μPAD only used glucose oxidase instead of conventional bienzymatic (GOx/peroxidase) device and it avoided bleaching of the final color, with a LOD of 5 µM. Some nanoparticles like graphene oxide (GO) and cerium oxide (CeO_2_) possessed high intrinsic peroxidase-like catalytic activity [103,104]. Deng et al. [105] synthesized GO@SiO_2_@CeO_2_ hybrid nanosheets (GSCs) as an alternative to the commonly employed peroxidase. 2,2′-azinobis(3-ethylbenzothiozoline)-6-sulfonic acid (ABTS) used as the electron donor dye substrate was converted from a colorless reduced form to a blue-green oxidized form by GSCs instead of HRP [106] with a LOD of 9 nM.

### 2.3. 3D-μPADs

Three-dimensional microfluidic paper-based analytical devices (3D-μPADs) represent an emerging platform development tendency due to the advantages of high throughput, complex fluid manipulation, multiplexed analytical tests, and parallel sample distribution [107]. Compared to the 2D μPADs, 3D-μPADs showed the advantage of highly homogeneous coloration that covering all the surface of the paper reaction zones. Fluid can move freely in both the horizontal and vertical directions in a 3D-μPAD. Yoon groups [108,109], Costa et al. [110] and Lewis et al. [111] fabricated 3D-μPADs by stacking alternating layers of patterned paper and double-sided adhesive tape with holes. In the presence of H_2_O_2_ generated by GOx, the HRP converts 4-AAP and *N*-ethyl-*N*-(2-hydroxy-3-sulfopropyl)-3,5-dimethylaniline sodium salt monohydrate (MAOS) from colorless compounds to a blue form, which can be visualized in the detection zone. Digital camera from a smartphone was utilized to read the signal and the dynamic detection ranges from 0.3 to 0.8 mM [109]. Li et al. [112] integrated a minimally invasive microneedle with 3D-μPAD to create the one-touch-activated blood diagnostic system, which shows great potential in clinical application.

**Figure 6:**
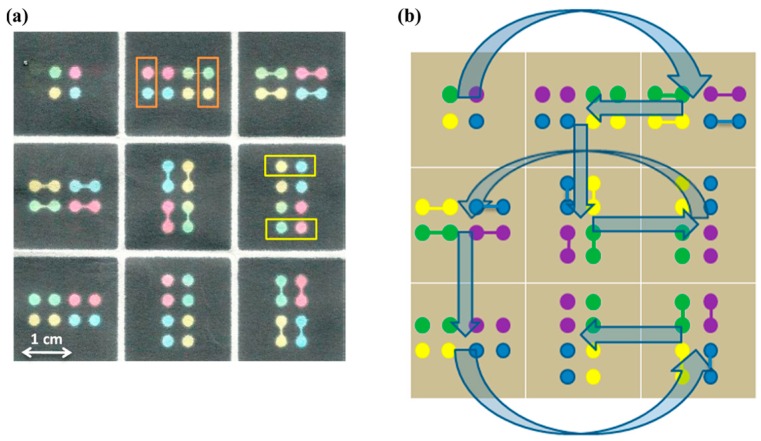
The paper-based 3D-μPADs designed in [115]. (**a**) The yellow rectangles of the unfolded μPADs marked out the protein detection region while the orange rectangles for glucose; (**b**) Flow pattern of the developed 3D-μPADs. Sample flow was introduced from the corner at the top left and spread into the middle square at the bottom layer. With the permission from [115]; Copyright 2013, American Chemical Society.

3D-μPADs could also be converted from 2D structures by origami [113,114,115]. Choi et al. [114] separated the 3D-μPADs into two layers. Reservoirs on the top layer were preloaded with reagent for glucose detection and the test solutions were loaded to each injection zone in the bottom layer. The device was used by tip-pinch manipulation with the thumb and index fingers to operate the chemical reaction of the preloaded reagent and test solutions. Sechi et al. [115] used 3D origami technique to fold the 3D-μPAD and the sample flows from the *x*, *y*, and *z* directions toward the detection points along the hydrophobic channels created by the wax printing technique (Figure 6).

**Figure 7:**
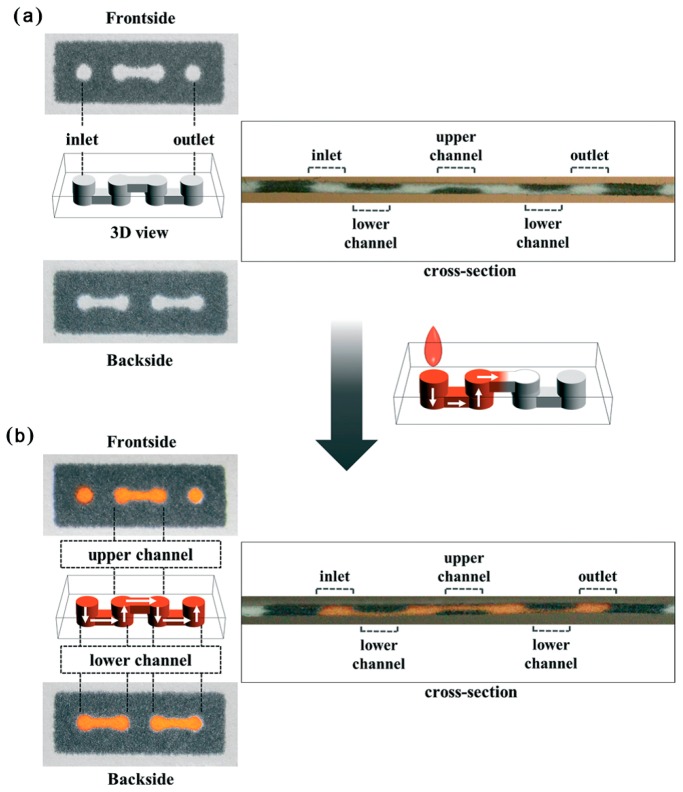
Scheme of the 3D-μPAD formation on a single sheet of paper in [116]. Before (**a**) and after (**b**) loading the red dye solution, the front, backside and cross section images of each parts indicated that the red dye solution had smoothly flowed from the inlet to the outlet via the alternative lower and upper channels. With the permission from [116]; Copyright 2015, The Royal Society of Chemistry.

Traditional fabrication techniques of 3D-μPAD involve stacking layers of patterned paper and origami-clamping, which are complicated and low efficiency. Li et al. [107] and Jeong et al. [116] proposed a method to fabricate a 3D-μPAD in a single layer of paper by doubled-sided printing and lamination (Figure 7). Through adjusting the density of printed wax and the heating time, penetration depth of melted wax could be controlled. This method eliminates major technical hurdles related to the complicated and interminable stacking, alignment, bonding and punching process.

The LODs achieved versus the colorimetric specific indicators through enzymatic reactions and the kinds of barriers explored were summarized in Table 1.

## 3. Electrochemical Detection of Glucose

### 3.1. Advanced Fabrications of Electrochemical Glucose μPADs

Electrochemical detection integrated with a paper-based analytical device plays an important role in glucose detection due to the advantage of low cost, high sensitivity and selectivity, minimal sample preparation and short time of response.

Screen-printed electrode (SPE) has been used for glucose detection in many paper-based analytical devices due to the advantage of flexible design and easy modification with chemicals. The research group of Swee Ngin Tan developed a paper-based amperometric glucose biosensor by placing a paper disk immobilized with glucose oxidase (GOx) on top of the SPE and used Fc-COOH or Prussian Blue (PB) as mediator [117,118]. The linear response range was 1–5 mM with a correlation coefficient of 0.971. The PAD showed a LOD of 0.18 mM. Yang et al. [65] modified the SPE with platinum nanoparticles (PtNPs) and used the enzymeless PtNPs-SPE to detect glucose oxidase reaction product H_2_O_2_. The detection limit was dropped to 9.3 μM. Noiphung et al. [119] added a plasma isolation part and used the PAD to detect glucose from whole blood. A polyvinyl alcohol-bound glass fiber was used to separate whole blood and the linear calibration range was from 0 up to 33.1 mM with a correlation coefficient of 0.987. Dias et al. [120] developed a paper-based enzymatic device to detect glucose in the 3D batch injection analysis (BIA) cell coupled with SPEs. The LOD was 0.11 mM and linear range was 1–10 mM. Miki et al. [121] replaced screen-printed electrode with complementary metal–oxide–semiconductor (CMOS) chips for electrochemical paper-based glucose detection. Electrodes were fabricated on CMOS chips, the working electrode (WE) and counter electrode (CE) were dropped with carbon ink, and the reference electrode (RE) was formed using Ag/AgCl ink. Glucose oxidase and electron mediator K_3_[Fe(CN)_6_] were immobilized on chromatography paper. Anodic currents given by electrodes were proportional to the glucose concentrations and linearity is up to 10 mM, which is sufficient for clinical applications [6].

### 3.2. Electrochemical Glucose μPADs with Printed Electrodes

**Figure 8:**
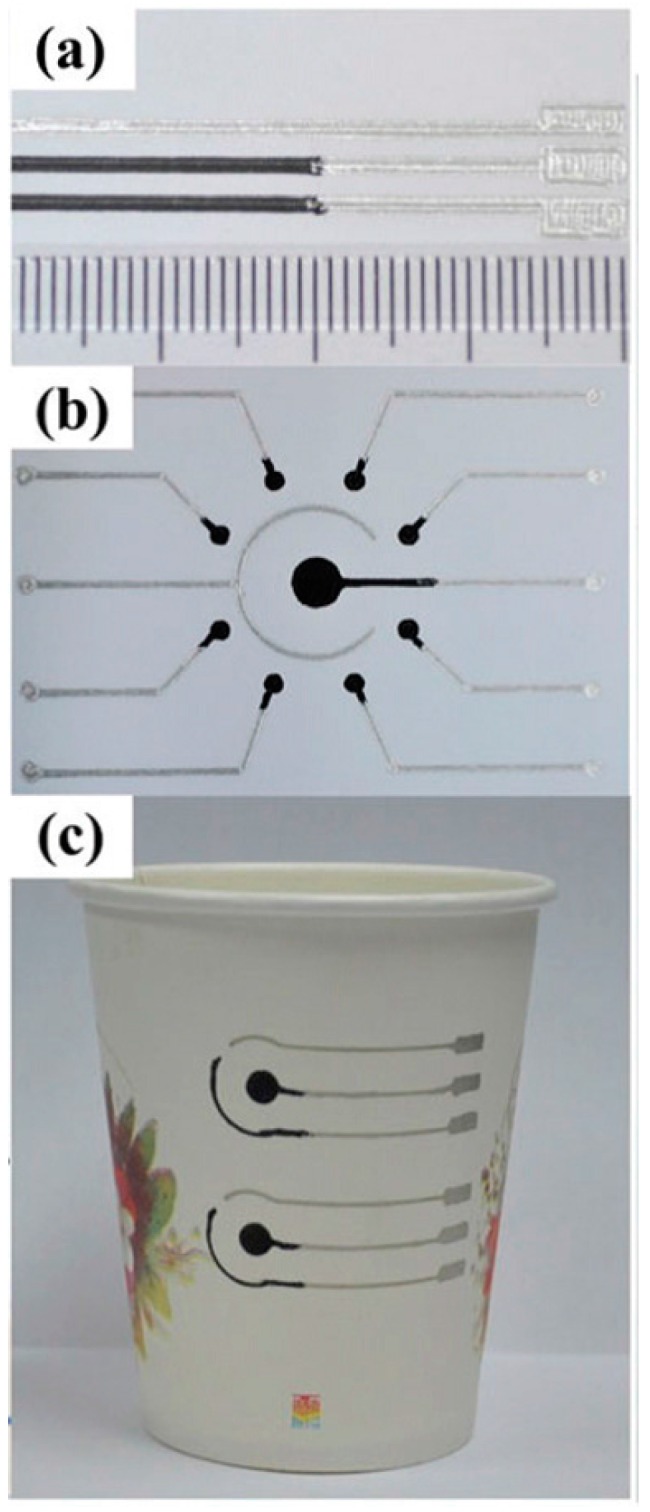
Photographs of electrochemical PADs built up on: A4 papers (**a**,**b**); and paper cups (**c**). Adapted with the permission from [123]; Copyright 2015, The Royal Society of Chemistry.

An electrochemical sensor is composed of substrate and electrode so that it is important to fabricate electrodes on paper using an easy and versatile method. Some scientists directly printed electrodes on paper substrate instead of using commercial screen-printed electrodes [66,67,68,122,123,124]. Rungsawang et al. [122] used 4-aminophenylboronic acid (4-APBA) as redox mediator to improve the selectivity of the homemade screen-printed carbon electrode due to the low detection potential and the detection limit was 0.86 mM. Määttänen et al. [67] used an inkjet-printing paper-based device, whose working and counter electrodes were printed gold-stripes and a silver-stripe was printed onto an AgCl layer to form the reference electrode. Several modifications were carried to demonstrate the inkjet-printing paper-based device showed no difference with conventional electrodes. Li et al. [123] proposed a direct writing method using a pressure-assisted accessory ball pen to fabricate electrodes on paper (Figure 8). The electrodes fabricated on paper were demonstrated with great electrical conductivity and electrochemical performance, and the electrode could be used in the artificial urine samples, which exhibited the potential in practical application. Li et al. [66] developed a three-electrode system prepared on paper directly by drawing with graphite pencils. The μPAD was designed with a sandwich-type structure that mediator and glucose oxidase were immobilized on separated zones. This origami μPAD showed acceptable reproducibility and high selectivity against interferents in physiological fluids. The linear calibration range was from 1 up to 12 mM and the LOD was 0.05 mM. Santhiago et al. [125] developed a dual-electrode system to replace the conventional three electrode systems. Graphite pencil was directly used as the working electrode instead of drawing on the paper. 4-aminophenylboronic acid was added as redox mediator to reach low limits glucose detection with a LOD of 0.38 μM.

The LODs achieved versus the electrochemical specific mediators through enzymatic reactions and the kinds of electrodes explored were summarized in Table 2.

## 4. Other Glucose Detection Platforms

Except for the conventional colorimetric and electrochemical techniques for glucose detection, there are some other techniques, such as luminescence [126], fluorescence [127], calorimetric [128], mass spectrum (MS) [129] and surface-enhanced Raman spectroscopy (SERS) [130] applied to μPADs for rapid glucose diagnostics. Chen et al. [126] developed a turn-on paper-based phosphorescence device using Ir-Zn_e_, a kind of luminescence sensing material, composited with GOx with layer-by-layer technique. Once glucose existed, the oxygen content was depleted and the phosphorescence of Ir-Zn_e_ increased concomitantly. The linear calibration range was from 0.05 to 8.0 mM with a correlation coefficient of 0.9956 and the LOD was 0.05 mM. Durán et al. [127] utilized colloidal CdSe/ZnS quantum dots (Q-dots) to produce an optical paper-based device for glucose detection. Paper loaded with Q-dots would display strong fluorescence under a UV lamp. H_2_O_2_ generated by GOx could cause fluorescence intensity to be quenched after a 20 min exposure. Calorimetric detection is demonstrated as an extension of current detection mechanisms of colorimetric and electrochemical μPADs. Davaji et al. [128] developed a calorimetric μPAD based on binding temperature of glucose/GOx for glucose detection through change in heat. Colletes et al. [129] presented a new insert sample method based on paper with paraffin barriers (PS-PB) and it was employed to glucose detection with a LOD of 2.77 mM. A paper membrane-based SERS platform was developed by Torul et al. [130] for glucose determination in blood using a nitrocellulose membrane and wax-printing microfluidic channel. Gold nanoparticles modified with 4-mercaptophenylboronic acid (4-MBA) and 1-decanethiol (1-DT) molecules were used as probe for μPADs. Glucose molecules were moved through the channel toward the measuring area constructed by dropping AuNPs on the membrane. The glucose concentration was 6.17 ± 0.11 mM and the device may provide a wide range of applications in daily life.

## 5. Conclusions

Rapid and convenient tests for glucose have become essential in underdeveloped and developing countries, as glucose is an important indicator of metabolic activity. Since microfluidic paper-based analytical device was proposed by the Harvard group in 2007, it has attracted extensive attention in a wide range of applications. Numerous methods have been developed to fabricate the μPADs and multiple detection techniques have been applied to glucose diagnostics. Colorimetric and electrochemical detection are doubtlessly the most important techniques. Colorimetric detection is more widely used than electrochemical detection while the sensitivity is lower than the latter. With the development of point-of-care diagnostic (POCT), it is expected that the carry-on paper-based analytical devices will be generated. The devices tend to be miniaturization and the spectrometric functions or electronic measurements could be integrated in the smartphones [131]. Alternative materials like toner [132,133] have also been investigated for clinical glucose diagnostics without the part of cumbersome fabrication process. Besides, the exploration of biocompatibility and toxicity of papers give a potential for developing minimally invasive or non-invasive μPADs for real-time glucose detection. Improvements of stability and accuracy of glucose detection will bring the devices to be commercially available in the future.

## Figures and Tables

**Table 1:** Summary of the colorimetric μPADs for glucose detection in representative references. TMB: 3,3′,5,5′-tetramethylbenzidine.

Reference	Indicator	Barrier	Limits of Detection
Garcia et al. (2014) [62]	KI	Paraffin	0.1 mM
Cai et al. (2014) [59]	KI	TMOS	Not mentioned
Li et al. (2016) [86]	KI	Wax	Not mentioned
Mohammadi et al. (2015) [88]	KI	PDMS	5 mM
Oyola-Reynoso et al. (2015) [92]	KI	Trichloro silane	5.5 mM
Chen et al. (2012) [4]	4-AAP/TOPS	Paper pieces	0.21 mM
Zhu et al. (2014) [94]	4-AAP/TBHBA	Paper pieces	0.3 mM
Zhou et al. (2014) [61]	APTMS/GA	Paper pieces	0.25 mM
Soni et al. (2015) [97]	Methyl red	Paper pieces	1.23 mM
Cate et al. (2013) [35]	DAB	Paper pieces	1.11 mM
Figueredo et al. (2016) [99]	TMB	Paper pieces	0.043, 0.062 and 0.018 mM (with different nanomaterials)
Palazzo et al. (2012) [98]	AuNPs	Paper pieces	0.1 mM
Deng et al. (2014) [105]	ABTS	Paper pieces	9 nM
Im et al. (2016) [109]	4-AAP/MAOS	Wax	0.3 mM
Gabriel et al. (2016) [96]	4-AAP/DHBS	Paraffin	0.023 mM

**Table 2:** Summary of the electrochemical μPADs for glucose detection in representative references. FcA: Ferrocenecarboxylic acid.

Reference	Mediator	Electrode	Limits of Detection
Lawrence et al. (2014) [118]	Fc-COOH	Commercial SPE	0.18 mM
Sekar et al. (2014) [117]	PB	Commercial SPE	0.01 mM
Yang et al. (2014) [65]	PtNPs	Commercial SPE	0.0093 mM
Miki et al. (2014) [121]	K_3_[Fe(CN)_6_]	CMOS	1 mM
Rungsawang et al. (2016) [122]	4-APBA	Wax-printing electrode	0.86 mM
Li et al. (2015) [123]	K_3_[Fe(CN)_6_]	Writing electrode	2 mM
Li et al. (2016) [66]	FcA	Graphite drawing electrode	0.05 mM
Santhiago et al. (2013) [125]	4-APBA	Graphite dual-electrode	0.38 μM
